# Solvent-Free Processing of Drug-Loaded Poly(ε-Caprolactone) Scaffolds with Tunable Macroporosity by Combination of Supercritical Foaming and Thermal Porogen Leaching †

**DOI:** 10.3390/polym13010159

**Published:** 2021-01-04

**Authors:** Víctor Santos-Rosales, Inés Ardao, Leticia Goimil, Jose Luis Gomez-Amoza, Carlos A. García-González

**Affiliations:** 1 Department of Pharmacology, Pharmacy and Pharmaceutical Technology, I + D Farma Group (GI-1645), Faculty of Pharmacy and Health Research Institute of Santiago de Compostela (IDIS), Universidade de Santiago de Compostela, E-15782 Santiago de Compostela, Spain; victor.santos.rosales@rai.usc.es (V.S.-R.); leticia.goimil@usc.es (L.G.); joseluis.gomez.amoza@usc.es (J.L.G.-A.); 2 BioFarma Research Group, Centro Singular de Investigación en Medicina Molecular y Enfermedades Crónicas (CiMUS), Universidade de Santiago de Compostela, E-15782 Santiago de Compostela, Spain; ines.ardao@usc.es

**Keywords:** scaffolds, supercritical CO_2_, foaming, solvent-free processing, ketoprofen, porogen

## Abstract

Demand of scaffolds for hard tissue repair increases due to a higher incidence of fractures related to accidents and bone-diseases that are linked to the ageing of the population. Namely, scaffolds loaded with bioactive agents can facilitate the bone repair by favoring the bone integration and avoiding post-grafting complications. Supercritical (sc-)foaming technology emerges as a unique solvent-free approach for the processing of drug-loadenu7d scaffolds at high incorporation yields. In this work, medicated poly(ε-caprolactone) (PCL) scaffolds were prepared by sc-foaming coupled with a leaching process to overcome problems of pore size tuning of the sc-foaming technique. The removal of the solid porogen (BA, ammonium bicarbonate) was carried out by a thermal leaching taking place at 37 °C and in the absence of solvents for the first time. Macroporous scaffolds with dual porosity (50–100 µm and 200–400 µm ranges) were obtained and with a porous structure directly dependent on the porogen content used. The processing of ketoprofen-loaded scaffolds using BA porogen resulted in drug loading yields close to 100% and influenced its release profile from the PCL matrix to a relevant clinical scenario. A novel solvent-free strategy has been set to integrate the incorporation of solid porogens in the sc-foaming of medicated scaffolds.

## 1. Introduction

Ageing of the population represents a major concern for the sustainability of the healthcare system. In Europe, the proportion of elderly people (aged 65 years and over) is expected to increase from 19 to 29% by 2070, coupled with a severe rise of 12% for people aged 80 and above [1]. At the same time, a drop of the working-age population of ca. 10% is prospected, thus representing a moderate share of the entire population. New biomedical technologies and approaches are thus requested to prompt longer working lives and healthier workers to mitigate the ageing burden. Namely, accidental and bone-diseases related fractures have a particular impact in the wellness and mobility of elderly people, leading to devastating physical and mental consequences that preclude the possibility of recovering the preceding welfare status of the patient [2].

Bone is the second most common transplantation tissue and its current gold-standard surgical procedure (implantation of biological grafts) is not exempt from clinical complications like immune rejection, risk of infections, and sequelae [3,4]. The advent of the regenerative medicine field facilitated the development of synthetic biodegradable grafts, known as scaffolds, offering a promising outlook for the full recovery of bone functionality. Scaffolds have been prepared from many biomaterials, including metals, ceramics, and a portfolio of biocompatible polymers [5,6]. The choice of biodegradable polymers is of preference since the response of the scaffolds to the biological environment favors gradual degradation rates of the scaffolds aiming to match the tissue regeneration tempos. In addition, the incorporation of bioactive agents (e.g., anti-inflammatory drugs and growth factors) to the synthetic grafts can improve the integration of the scaffolds to the surrounding biological environment and mitigate post-surgical comorbidities such as excessive inflammation, pain, or the risk of infections [7,8,9,10,11].

Among the manufacturing techniques for the preparation of polymeric scaffolds, the supercritical CO_2_-assisted foaming, the so-called supercritical (sc-) foaming, offers significant advantages regarding the solvent-free processability of materials and the loading of bioactive compounds in an integrated process and at high yields [12]. The sc-foaming technique relies on two main sequential steps: (1) CO_2_ sorption in the polymeric matrix under a target working temperature and pressure, and (2) controlled pressure reduction with pore formation. Porosity is generated in these structures through a nucleation-growth mechanism taking place due to the sudden supersaturation of CO_2_ in the polymer as the pressure decreased. The CO_2_ escape from the matrix leads to the vitrification of the polymer, stabilizing the formed porous structure [13]. The pressure-dependent plasticizing effect of CO_2_ allows the processing of polymers under mild temperatures, thus unlocking the possibility of an in situ incorporation of thermolabile compounds, such as growth factors, with low degradation and activity losses [14]. Moreover, the safety of use of carbon dioxide (low toxicity) and its recyclability makes it a green technology with the associated environmental benefits.

The preparation of scaffolds using the sc-foaming method allows the tuning of their porous morphology (porosity, mean pore size, and pore size distribution) to fit certain target specifications [15]. Processing temperature and pressure, CO_2_ contact time and depressurization rate are the main foaming parameters able to adjust the pore size and homogeneity [16,17]. However, the limitations of sc-foaming are related to the complex modelling of the pore formation mechanisms to get a precise control and predictability of the pore sizes and distributions obtained for the processed scaffolds. Moreover, the use of depressurization gradients [18] and leaching methods with particulate porogens (e.g., NaCl, sucrose, carbonates, bicarbonates, zein [19,20,21,22,23,24,25,26]), and sacrificial polymers [27] within this foaming process can result in scaffolds with more open porosity and dual macroporosity (i.e., porous materials with two pore families in the macroporous range) [19,28]. The use of porogens is the most suitable approach to reach an additional macropore family with well-defined porosity and narrow pore size distribution, although an extra processing step will be usually needed to remove the porogen by solvent (usually water) leaching and the advantageous solvent-free property of sc-foaming technique is thus omitted [12,29]. In the case of drug-loaded scaffolds, the leaching of the bioactive agent contained in the scaffold formulations may take place along with the porogen removal resulting in dramatic reductions in drug incorporation yields [29,30]. Overall, novel strategies are needed to align the incorporation of solid porogens in the sc-foaming of medicated scaffolds.

Medicated scaffolds obtained by sc-foaming and using a porogen that can be removed without any solvent leaching process involved were herein produced. In this work, scaffolds medicated with drugs were prepared by sc-foaming using ammonium bicarbonate (AB) porogen. AB was the selected porogen since it can be removed through thermal degradation at temperatures as low as 35–40 °C [31,32], compatible with thermally sensitive compounds. Ketoprofen, a nonsteroideal anti-inflammatory drug (NSAID) commonly used for pain relief and to reduce inflammation occurring post-implantation [33], was herein selected as model drug within the scaffold formulation for local administration. This local treatment will avoid the common gastrointestinal disorders linked to the systemic ketoprofen administration by oral delivery [34]. The effect of this processing strategy on the physicochemical properties of the scaffolds, on the drug loading yield and on cytocompatibility was evaluated. Finally, the effect of the use of this porogen on the release profiles of the ketoprofen-loaded polymeric scaffolds was determined. To the best of our knowledge, this is the firstly reported solvent-free approach incorporating the use of solid porogen particles to induce a new macropore population in scaffolds obtained by sc-foaming.

## 2. Results and Discussion

### 2.1. Scaffolds Development and Morphological Characterization

**Figure 1:**
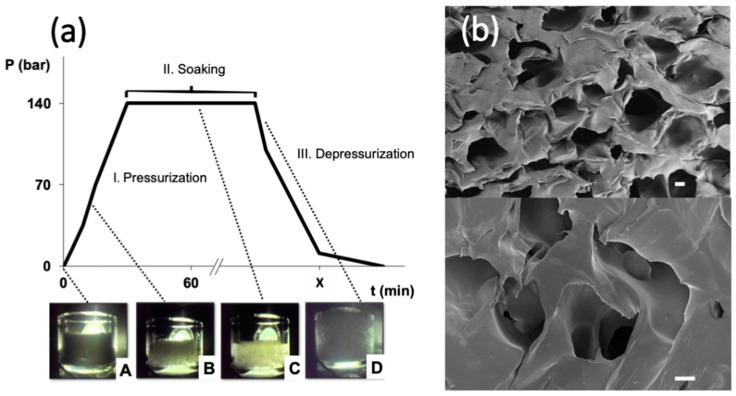
Development of poly(ε-caprolactone) (PCL) scaffolds through the sc-foaming method: (**a**) Typical pressure (P) vs. time (t) profile of sc-foaming tests performed in this work with PCL scaffolds. For certain cases, a visual inspection (pictures at the bottom) of the physical phenomena taking place during the sc-foaming steps (I. pressurization, II. Soaking, and III. depressurization) was carried out: (A) raw PCL powder before CO_2_ pressurization, (B) CO_2_ solubilization in PCL, (C) polymer melting, and (D) polymeric expansion and formation of pores. (**b**) SEM images of a PCL scaffold processed by sc-foaming at 37 °C and 140 bar with a soaking time of 1 h and slow depressurization (3 bar/min). Scale bars: 100 μm.

Tissue regeneration requires scaffolds with a suitable porous structure, usually in the 60–80% porosity range and with interconnected macropores of sizes of 75 µm and above to facilitate cell colonization and growth [35]. The ultimate goal of the scaffold is to promote the generation of a mature biological tissue after a certain post-implantation period (in the order of months or even years). These specific porous features can be achieved in thermoplastic polymers through the sc-foaming process, which is assisted by the plasticizing effect of compressed CO_2_. In the case of the poly(ε-caprolactone) (PCL) used in this work, the biopolymer had a melting point depletion upon contact with pressurized CO_2_ at 140 bar of more than 20 °C with respect to its normal melting point (61.4 °C). Accordingly, molten PCL polymer was obtained at 37 °C in a supercritical CO_2_ atmosphere after a certain time period (point C in Figure 1a). This low processing temperature used in the sc-foaming technique is unbeatable by any other alternative thermal-based foaming method (e.g., melt-molding, melt extrusion, and fused deposition modelling).

During the depressurization stage of the sc-foaming process, CO_2_ is vented out of the polymeric structure in a controlled way leading to PCL vitrification and the formation of a macroporous foam (point D in Figure 1a,b). Pore size of the scaffolds can be modulated up to a certain extent by the depressurization rate with lower values favoring the formation of larger pores [16,36,37,38]. Using this approach, PCL scaffolds with high porosity (63%) presenting macropores with smooth surfaces and 80% interconnectivity were obtained (Figure 1b and Table 1). Overall, sc-foaming technology opens up new processing possibilities to obtain scaffolds at low operating temperatures allowing the feasibility of the production of polymeric foams incorporating thermally-sensitive bioactive agents.

The compatibility of the scaffold processing method used with the incorporation of bioactive compounds in the scaffold formulations was tested using ketoprofen, a hydrophobic NSAID with high affinity to the PCL matrix. The drug loading yield was close to 100% since the remaining weight of the scaffold after the foaming process corresponded to the sum of the initial weights of PCL and ketoprofen. The addition of ketoprofen resulted in a densification of the scaffolds (PCL(5K)0BA scaffold in Table 1) with lower porosities (54%) and pore interconnectivity (65%). The melting point of the PCL was reduced in the presence of ketoprofen (Table A1). This effect was related to PCL-ketoprofen chemical interactions and not to the processing method as this variation in the melting event was also observed after the second DSC-heating cycle [39].

The use of BA porogen in the sc-foaming of medicated scaffolds and their subsequent removal by a leaching process in the absence of liquid solvents was tested. The elimination of this porogen was carried out at 37 °C and under vacuum to favor the thermal degradation of BA into gaseous ammonia and carbon dioxide and at temperatures well below the normal melting point of the PCL [20,29,40]. The thermal leaching process used in this work for porogen removal would then surrogate the conventional solvent-based leaching process. The removal of the porogen by vacuum heating just after the foaming process was complete after a 10-day treatment according to the observed scaffold weight losses (data not shown). The use of vacuum accelerated the porogen degradation in more than four days with respect to the use of atmospheric drying. After this thermal post-processing, the melting point of PCL was almost unaltered (61–62 °C) and its crystallinity increased from 60% to 71–75% with respect to PCL scaffolds obtained by the same foaming conditions and without thermal post-treatment (Table A1). BA might act as a secondary nucleation site for PCL crystallization during the sc-foaming process as observed with other particle admixtures [14].

The incorporation of BA as a porogen in the scaffold formulation assisted the sc-foaming process to obtain scaffolds with customized porosity (Table 1). The use of porogen in 50 and 75 wt.% of the total initial mixture formulation resulted in dramatic increases of the overall porosities in ca. 15 and 18%, respectively. The increase in the porosity of the scaffolds with a higher BA content was also observed with scaffolds processed by phase inversion followed by leaching of BA [29,40]. The maximum possible content of BA in the formulations was set at 75% since higher porogen contents resulted in very brittle porous materials.

**Figure 2:**
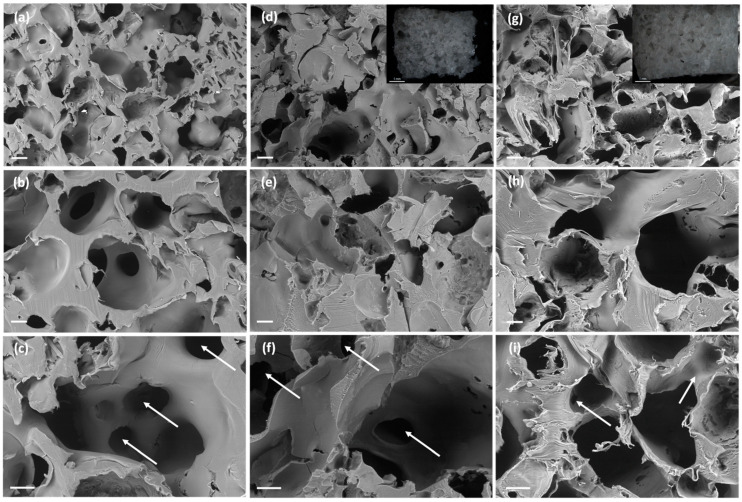
SEM-images of cross sections of (**a**–**c**) PCL(0K)50BA, (**d**–**f**) PCL(5K)50BA, and (**g**–**i**) PCL(10K)50BA polymeric scaffolds loaded with 0, 5, and 10 wt.% of ketoprofen, respectively. Insets in (**d**,**g**): Optical micrographs of the scaffolds. The presence of interconnected pores was observed for all the formulations (white arrows in **c**,**f**, and **i**). Scale bars: (**a**,**d**,**g**) 200 µm; (**b**,**c**,**e**,**f**,**h**,**i**) 100 µm; (insets) 1 mm.

**Figure 3:**
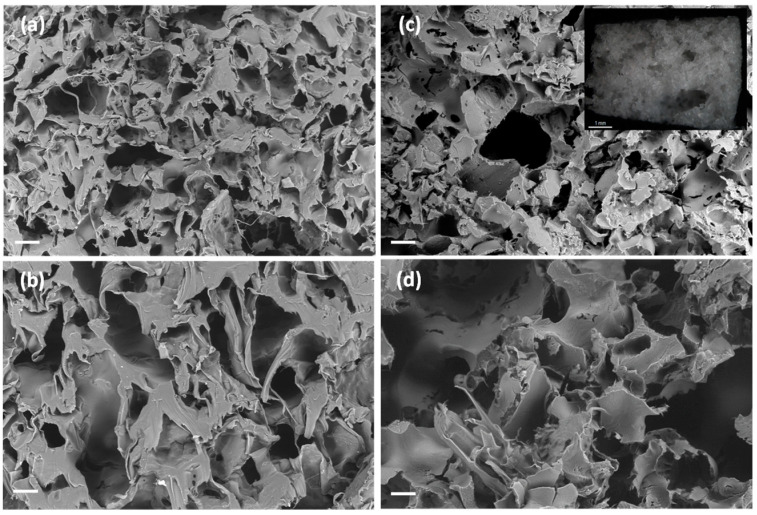
SEM-images of cross sections of (**a,b**) PCL(0K)75BA and (**c,d**) PCL(5K)75BA polymeric scaffolds loaded without and with 5 wt.% of ketoprofen, respectively. Insets in (**a**,**c**): Optical micrographs of the scaffolds. Scale bars (**a**,**c**): 200 µm; (**b**,**d**) 100 µm; 1 mm (inset).

SEM imaging unveiled a change of porous morphology with the use of BA porogen consisting on the presence of a second pore family of large macropores in the 200–400 µm size range, i.e., of similar size to the porogen size (Figure 2a–c), along with a pore family in the 50–100 µm size range related to the plasticizing and porogenic effects of compressed CO_2_. This new macroporous family falls in the optimum pore size of 325 µm for bone tissue engineering, where the lower cell adhesion with respect to smaller pores is compensated by an enhanced cell migration [35,41]. No remnants of BA particles were observed in the SEM pictures confirming the full porogen removal. Interestingly, the scaffolds presented interconnected pores through throats in the order of 50–150 µm (white arrows in Figure 2c,f,i). Ketoprofen was fully incorporated in the polymeric matrix of the scaffolds and drug crystals were not observed. Ketoprofen can be molecularly dispersed or in the amorphous form within the PCL-based scaffolds [42]. The presence of ketoprofen did not interfere with the pore formation process upon sc-foaming and similar porous structures were obtained for the PCL scaffolds loaded with 5 and 10 wt.% of the drug (Figure 2d–i). Finally, the proportion of the family of large macropores in the 200–400 µm range increased with a higher BA content (Figure 3), thus supporting the origin of these pores due to the presence of the porogen.

**Figure 4:**
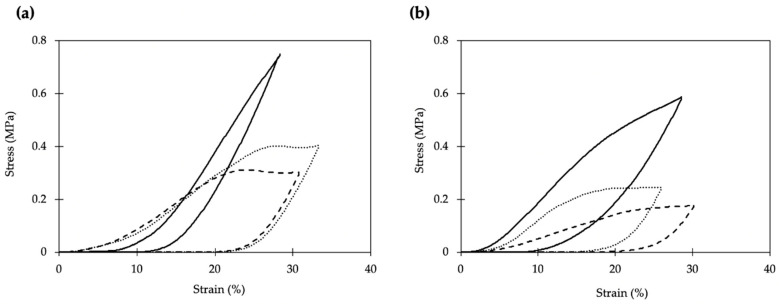
Stress-strain response under uniaxial compression of the manufactured scaffolds: (**a**) PCL50BA formulations, and (**b**) PCL75BA formulations with different drug (K) contents (0, 5, and 10 wt.%). Legend: black line (0 K); dotted line (5 K) and dashed line (10 K).

The mechanical properties of PCL scaffolds were tested under uniaxial compression tests (Figure 4). Young’s moduli were in the 1 to 5 MPa range, depending on the scaffold formulation, which is coherent for the intended application [43]. Higher porogen contents during the scaffold processing reduced the mechanical properties, and scaffolds processed with the highest BA content were significantly softer (Figure 4). The effect of porosity on the mechanical performance of the materials is highly dependent on the pore sizes. Pores in the microporous or low mesoporous range do not have a high effect on the mechanical properties of the material [44,45]. In the case of macroporous materials, they usually exhibit a power-law scaling relationship between Young’s modulus and the bulk density [46]. The induction of the new macropore population in the PCL scaffolds reduced the bulk density and, therefore, reduced the mechanical performance of the scaffolds.

Despite the ketoprofen incorporation had no impact on the morphology of the scaffolds, the viscoelastic behavior was remarkable modified (Figure 4). Regardless of the porogen content, scaffold formulations processed with ketoprofen presented plastic deformations of ca. 20% that are not observed in the unloaded scaffolds (black lines in Figure 4). The ketoprofen incorporation effect on the mechanical properties was consistent and also identified for formulations with higher porogen contents.

### 2.2. Ketoprofen Release Studies

**Figure 5:**
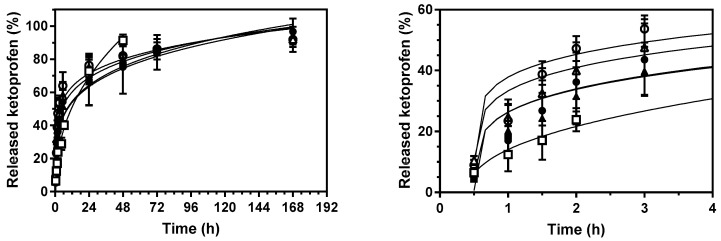
Ketoprofen release from PCL-based scaffolds in PBS pH 7.4 at 37 °C and 60 rpm during 7 days (**left**) and close up of the profiles during the first hours (**right**). Lines correspond to the fitting of the experimental data to the Korsmeyer-Peppas with lag time drug release model (Equation (2)). Legend: PCL(5K)0BA (white squares), PCL(5K)50BA (black triangles), PCL(10K)50BA (white triangles), PCL(5K)75BA (black circles), and PCL(10K)75BA (white circles).

The effect of ketoprofen entrapment into the PCL scaffolds by sc-foaming coupled to the effect of porogen content on the capacity of modulation of the drug release kinetics were evaluated in PBS pH 7.4 medium (Figure 5). After a lag time of ca. 30 min, all the tested ketoprofen-loaded scaffolds provided a fast initial release, reaching ca. 70% of dissolved ketoprofen at 24 h, followed by a more sustained release in the following days. Ketoprofen release was stabilized after one week reaching a drug payload above 90% in all cases. Complete drug release was obtained after three weeks for all the formulations. The drug contents dissolved in PBS medium after three weeks matched those of the initial drug content in the formulations before the foaming-plus-leaching process, thus confirming ketoprofen loading yields in the scaffolds close to 100%. The release profile of these medicated scaffolds is of clinical relevance since it would provide anti-inflammatory responses during the bone healing process through a fast action in the first hours after implantation and keeping a certain drug local concentration at least during one week [47]. Moreover, the ketoprofen content per scaffold (65–130 mg) falls in-between the approved doses (200 mg) upon oral administration for systemic delivery and the doses in patches (30–100 mg) for local delivery [34] The obtained versatility in ketoprofen content within the sc-foamed bone scaffolds opens up the possibility of delivering the required doses to bone defects of different sizes.

The addition of the porogen during the scaffold processing had a significant effect on the drug release kinetics (Figure 5). Scaffolds processed in the presence of porogen had a faster drug release kinetics (Figure 5, right). This effect was even more pronounced if the porogen-to-PCL weight ratio was increased from 1:1 to 3:1, regardless of the drug content used (5 or 10 wt.%). In general, scaffold porosity and pore size distribution are among the main factors influencing the release profiles of drugs in medicated scaffolds [42,48]. The higher porosities and larger pore sizes obtained for the scaffolds processed with porogens favor the accessibility of the aqueous fluid medium throughout the porous structure of the scaffold, thus accelerating the drug release, notably during the first hours.

Drug release profiles were fitted to the Korsmeyer-Peppas with lag time equation model (Equation (2)) with good correlation levels (Table 2). The kinetic coefficient *k* was significantly increased in the presence of porogen, confirming the influence of BA in accelerating the drug release. Interestingly, *k* was much higher for scaffolds with higher ketoprofen contents (PCL(10K)50BA and PCL(10K)75BA). The use of porogen did not have an influence in the initial release time (ca. 0.50 h), since similar t_lag_ values were obtained for scaffolds processed with different BA contents and even in the absence of the porogen (0.45 h). *n* values were below 0.45 in all cases suggesting a complex diffusion-controlled release mechanism of the drug through the polymeric matrix and the pores filled with the PBS medium [14,49]. The kinetics of ketoprofen-loaded scaffolds of PCL using BA porogen were much faster than that reported for PCL-scaffolds prepared by sc-foaming with starch aerogel microparticles as porogen and at the same drug content [42]. The faster release with BA porogen was related to a higher porosity and mean pore size with respect to the use of starch aerogel porogen.

### 2.3. PCL-Scaffold Degradation Studies

**Figure 6:**
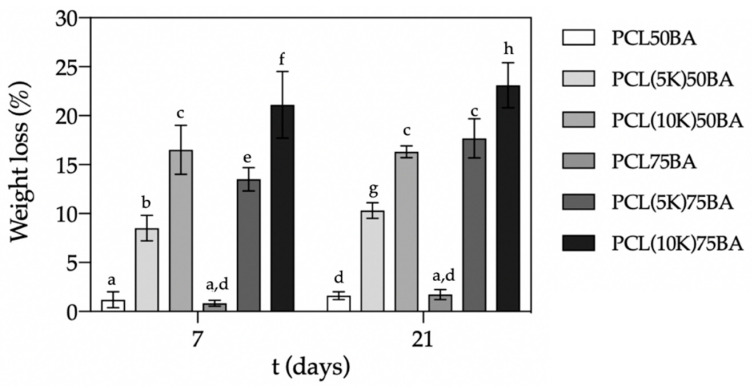
Weight losses of PCL scaffolds after 7 and 21 days in PBS pH 7.4 medium at 37 °C and 60 rpm. Equal letter denotes statistically homogeneous groups (1-way ANOVA; *p* < 0.05).

Scaffolds were evaluated regarding their porous structures and weight losses after 21 days in PBS pH 7.4 medium. Weight losses in the scaffolds were higher than those assigned to the release of the drug payload (Figure 6). This discrepancy in weight was related to PCL degradation and erosion and was higher for PCL(10K)50BA and PCL(10K)75BA scaffolds with higher drug contents (10 wt.%). PCL erosion takes place through hydrolytic chain scission of the ester groups and the process is favored with higher porosities [50,51].

**Figure 7:**
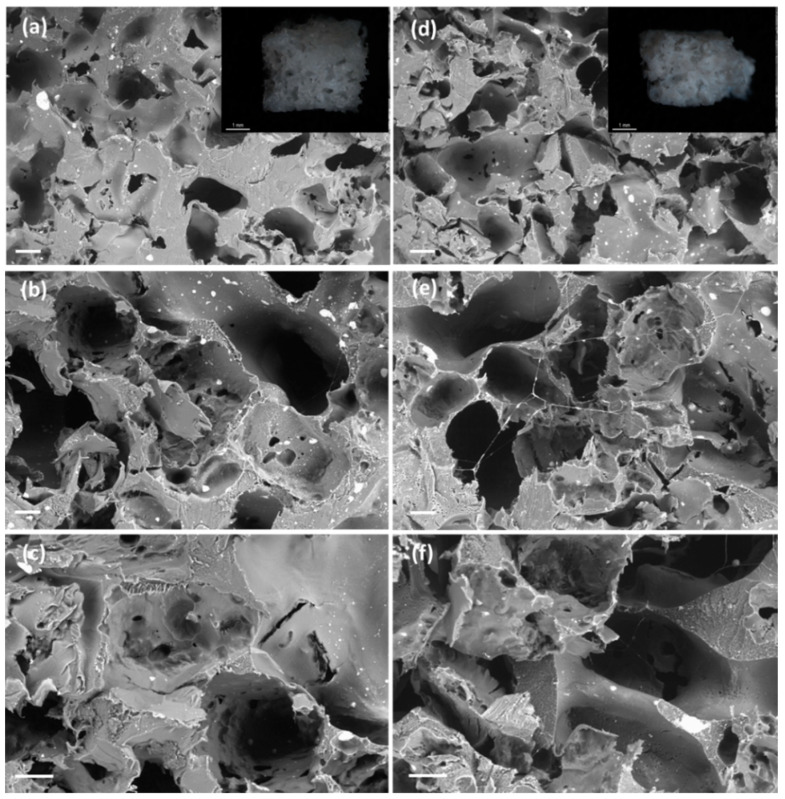
SEM-images of cross sections of (**a**–**c**) PCL(5K)50BA and (**d**–**f**) PCL(10K)50BA polymeric scaffolds loaded with 5 and 10 wt.% of ketoprofen, respectively, after 21 days in PBS pH 7.4 medium. Insets in (**a**,**d**): Optical micrographs of the scaffolds. Scale bars (**a**,**d**): 200 µm; (**b**,**c**,**e**,**f**) 100 µm; (insets) 1 mm.

**Figure 8:**
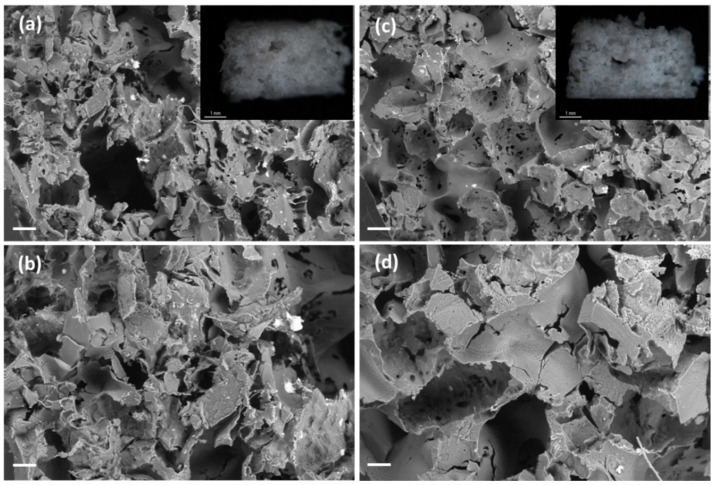
SEM images of cross sections of (**a**,**b**) PCL(5K)75BA and (**c**,**d**) PCL(10K)75BA polymeric scaffolds loaded with 5 and 10 wt.% of ketoprofen, respectively, after 21 days in PBS pH 7.4 medium. Insets in (**a**,**c**): Optical micrographs of the scaffolds. Scale bars (**a**,**c**): 200 µm; (**b**,**d**) 100 µm; (insets) 1 mm.

The porogen content had a significant impact (*p* < 0.05) in the weight losses of the scaffolds after 7 and 21 days, except for unloaded PCL scaffolds (Figure 5). For instance, scaffolds with the same ketoprofen content suffered an increased degradation of ca. 7% after 21 days when the porogen content was higher. The morphology, porous structure and surface roughness of the scaffolds were almost unaltered after the in vitro degradation test for three weeks (Figure 7 and Figure 8). Moreover, PCL-based scaffolds might undergo faster degradation rates in vivo than under in vitro conditions [52].

### 2.4. Cytotoxicity Tests

**Figure 9:**
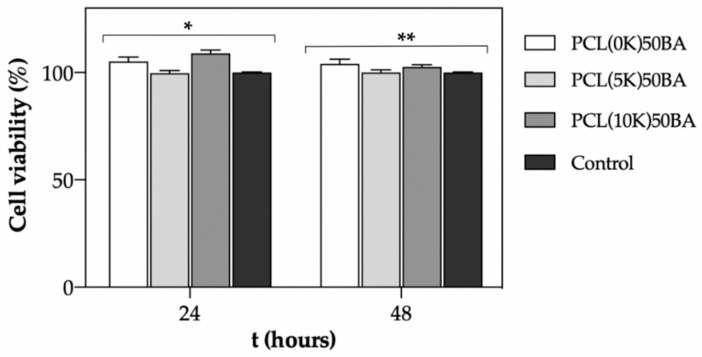
Fibroblast cell viability determined by CCK-8 test after 24 and 48 h of direct contact with the manufactured scaffolds. Viability was expressed in percentage. Equal symbol denotes statistically homogeneous groups (1 way-ANOVA; *p* < 0.05).

In vitro cell viability tests of murine fibroblasts in the presence of PCL-based scaffolds were evaluated in the formulations with the lower porogen contents (50 wt.%) due to their improved mechanical performance. After 24 h of incubation, all formulations presented excellent cytocompatibility with values close to 100% and similar to the control (*p* < 0.05) (Figure 9). Therefore, no remnants of BA were still in the polymeric matrix, since externally added ammonia is known to reduce the growth rate of mammalian cell lines even at low concentrations (2–3 mM). This phenomenon occurs through the disruption of intracellular and intraorganelle pH, ultimately perturbing electrochemical gradients or by direct interaction with enzymes [53]. Excellent cytocompatibility was also obtained after 48 h of direct incubation with the manufactured PCL-based scaffolds.

## 3. Materials and Methods

### 3.1. Materials

Poly(ε-caprolactone) (PCL; powder, Mw = 50 kDa, T_m_ = 61.4 °C, 66.7% crystallinity) was purchased from Polysciences (Warrington, PA, USA). Ammonium bicarbonate (BA; NH_4_HCO_3_; 30% minimum content in NH_3_) from Panreac (Castellar del Vallès, Spain) was used as porogen. Ketoprofen (K; T_m_ = 95.8 °C, 99.7% purity) was provided by Acofarma (Terrassa, Spain). CO_2_ (purity > 99.9%) was provided by Praxair, Inc. (Madrid, Spain) and used as foaming agent.

### 3.2. Macroporous PCL-Ketoprofen Composite Scaffold Preparation

Firstly, BA was sieved and the particles of size in the 250–500 µm range were collected for further use. Ketoprofen-loaded PCL-scaffolds were prepared from formulations with different PCL:BA:K weight ratios (Table 1). For each scaffold, 1.3 g of the initial components were dosed in cylindrical (length = 24.6 mm, inner diameter = 17 mm) Teflon molds (Brand GmbH, Wertheim, Germany), manually mixed using a spatula and further compacted with an aluminum plunger. Molds were then placed in a high-pressure autoclave (Thar Technologies, Pittsburgh, PA, USA) and subjected to the sc-foaming process through a pressurization-soaking-single depressurization protocol [42]. Briefly, the autoclave was filled with CO_2_ at the mass flow rate of 5 g/min until the target supercritical conditions of 37 °C and 140 bar were achieved. After 1 h in the static mode, the system was depressurized at a rate of 3 bar/min until atmospheric pressure.

The obtained scaffolds were collected and placed in a vacuum oven (W.C. Heraeus GmbH, Hanau, Germany) at 37 °C and 100 mmHg for porogen removal through thermal decomposition. Scaffolds were weighed periodically until their weight was constant, i.e., the porogen leaching process was complete. The same procedure but at atmospheric pressure was also tested for the sake of comparison. Drug-loaded scaffolds were denoted as PCL(*x*K)*y*BA, being *x* and *y* the ketoprofen and BA contents in weight percentage with respect to PCL content and to the total weight, respectively.

### 3.3. In Situ Visual Follow-Up of the Supercritical Foaming of Scaffolds

A borosilicate vessel (length = 12 mm, internal diameter= 16.5 mm) filled with 1 g of PCL was placed within the foaming autoclave and then the standard sc-foaming protocol (cf. Section 3.2) was carried out. A borescope endoscope (Flylink Technology Co., Shenzhen, Guangdong, China) was placed pointing at one of the sapphire windows of the foaming autoclave to visually follow-up the thermophysical events taking place within the autoclave through image sequences.

### 3.4. Structural and Physicochemical Characterization of the Scaffolds

The dimensions and weight of the scaffolds were measured after removal of the porogen to determine the bulk density (*ρ_bulk_*) from three replicates. The skeletal density (*ρ_skel_*) of the scaffolds was determined by helium pycnometry (Quantachrome, Boynton Beach, FL, USA) from six replicates and measured at 25 °C and 1.01 bar. Overall porosity values (*ε*) were obtained from the bulk and skeletal density values according to Equation (1).

(1) $$\varepsilon \;\left(\%\right)=\left(1-\frac{\rho_{bulk}}{\rho_{skel}}\right)\;\times \;100$$

The morphological properties of the scaffolds were assessed before and after the drug release studies (cf. Section 3.5) by digital imaging using a CCD Microscope Camera (DFC7000 T, Leica, Wetzlar, Germany) and scanning electron microscopy (SEM; EVO LS15, Zeiss, Oberkochen, Germany). Prior to imaging, scaffolds were cut with a scalpel.

Thermal properties of the scaffolds were analyzed by differential scanning calorimetry (DSC-Q100, TA Instruments, New Castle, DE, USA) under a nitrogen atmosphere with two heating cycles up to 200 °C under a rate of 10 °C/min with an intermediate cooling cycle down to −10 °C.

The mechanical behavior of PCL-scaffolds was analyzed through orthogonal compression tests using a 30 kg load cell (TA.XTPlus, Stable Micro Systems, Ltd., Godalming, UK) at a crosshead speed of 1 mm/min until a strain rate of ca. 30%. All the experiments were performed at 20 °C, atmospheric pressure and in duplicate.

### 3.5. Ketoprofen Release Studies

Scaffolds were cut in pieces of 10 mg after porogen removal and suspended in flasks containing 50 mL of PBS pH 7.4 solution as release medium. Release studies were performed under sink conditions (ketoprofen solubility in PBS, pH 7.4 and 37 °C = 2.2 mg/mL [54]). Flasks were placed in an oscillating bath (Unitronic 320 OR, Selecta, Barcelona, Spain) at 37 °C under an agitation of 60 rpm for 21 days. Aliquots (3 mL) were sampled from the release medium period at selected times. The withdrawn volumes were replaced in the medium with fresh PBS solution. Samples were filtered (0.2 mm nylon filters) and then the ketoprofen content in the solution was measured by UV–Vis spectrophotometry (8453, Agilent, Santa Clara, CA, USA) at the wavelength of 260 nm [42]. Ketoprofen standard solutions in PBS were performed at concentration ranging from 0.001 to 0.025 mg/mL (R^2^ > 0.999). Release tests were performed in triplicate. Once the release test was finished after 21 days, scaffolds were collected, washed gently with Milli-Q water, freeze-dried and observed by SEM.

Ketoprofen release data were fitted to the Korsmeyer-Peppas with lag time equation (Equation (2)) using GraphPad Prism v.6.04 for Windows software (GraphPad Software, La Jolla, CA, USA)

(2) $$F=k\cdot {\left(t-t_{lag}\right)}^{n}$$

where *F* is the fraction of drug released at a time *t*, *k* is a kinetic coefficient related to the macromolecular polymeric network structure, *t_lag_* is the lag time, and *n* is the diffusional exponent.

For the degradation studies, scaffolds (20 mg) were suspended in 1 mL of PBS pH 7.4 solution, collected after a certain time period (7 and 21 days), washed gently with Milli-Q water, freeze-dried and weighed.

### 3.6. Cytotoxicity Tests

Murine fibroblasts (CCL-163, ATCC, USA) were employed to evaluate the cytotoxicity of the manufactured scaffolds. Cells were seeded in 24-well plates (30,000 cells/well) with DMEM medium supplemented with 10 % FBS and 1 % penicillin (10,000 UI/mL)/streptomycin (10,000 µg/mL). Culture plate was maintained at 37 °C in a humidified atmosphere enriched with 5% CO_2_ for 6 h to allow the cell attachment to the bottom of the well.

Prior to seeding, cubic scaffold pieces were sterilized by soaking in EtOH 70% (*v*/*v*) for 3 min, followed by drying in a laminar flow cabinet at room temperature. Afterwards, scaffolds were incubated with cells in quadruplicate for 24 and 48 h at 37 °C in a humidified atmosphere with 5% CO_2_. Controls included cells incubated without material (negative control).

Cell proliferation was evaluated using the Cell Counting Kit-8 (CCK-8) (Roche, Basel, Switzerland) at 24 and 48 h and performed according to the manufacturer’s protocol. Absorbance was read at the wavelength of 450 nm (UV BioRad Model 680 microplate reader, Hercules, CA, USA). Cell viability (%) was calculated as follows:

(3) $$Cell\;viability\;\left(\%\right)=\;\frac{Abs_{exp}}{Abs_{negative\;control}}\;\times 100$$

### 3.7. Statistical Analysis

Statistical analyses (1-way ANOVA) of the weight loss after the degradation test and of the cell viability tests were performed using Statistica v8.0 software (StatSoft Inc., Tulsa, OK, USA) followed by the post hoc Tukey’s HSD multiple comparison test.

## 4. Conclusions

An innovative processing approach has been developed to obtain drug-loaded scaffolds by supercritical foaming coupled with solid porogen removal avoiding the critical step of solvent leaching of the said porogen along with the drug. Supercritical foaming technology opens up new processing possibilities to obtain scaffolds at low operating temperatures (37 °C). The use of ammonium bicarbonate as porogen allowed its removal from the polymeric scaffolds through thermal degradation at similar temperatures as the foaming process. The incorporation of BA as a porogen in the scaffold formulation resulted advantageous in obtaining a second pore family in the required pore size range for regenerative medicine purposes and assisted the supercritical foaming process to engineer scaffolds with dual porosity. This solvent-free technology offers a variety of possibilities of tuning porosity and pore sizes in medicated scaffolds through the porogen content and size, respectively. In addition, the PCL-based scaffolds were cytocompatible with murine fibroblast cells after 48 h of direct contact. Finally, the low processing temperature used allows the technical feasibility of the production of polymeric foams incorporating thermally-sensitive bioactive agents.

## Figures and Tables

**Table 1:** Composition (expressed in weight percentage) of the initial mixtures used for the scaffold formulations and textural properties of the resulting macroporous ketoprofen-loaded PCL scaffolds by sc-foaming combined with BA porogen leaching.

	Initial Formulation Composition			Textural Properties		
Scaffold	PCL (wt.%)	K (wt.%)	BA (wt.%)	ρ_bulk_ (g/cm^3^)	ρ_skel_ (g/cm^3^)	ε (%)
PCL(0K)0BA	100	-	-	0.41 ± 0.01	1.126 ± 0.015	63.6 ± 1.0
PCL(5K)0BA	95.0	5.0	-	0.51 ± 0.08	1.098 ± 0.004	53.6 ± 7.3
PCL(0K)50BA	50.0	-	50.0	0.24 ± 0.01	1.110 ± 0.011	78.4 ± 0.5
PCL(5K)50BA	47.4	2.6	50.0	0.29 ± 0.04	1.032 ± 0.006	71.9 ± 3.4
PCL(10K)50BA	47.4	5.3	50.0	0.28 ± 0.07	1.023 ± 0.009	72.6 ± 4.4
PCL(0K)75BA	25.0	-	75.0	0.20 ± 0.01	1.086 ± 0.006	81.6 ± 0.6
PCL(5K)75BA	23.7	1.3	75.0	0.18 ± 0.05	1.047 ± 0.005	82.8 ± 3.6
PCL(10K)75BA	22.4	2.6	75.0	0.18 ± 0.07	1.034 ± 0.007	82.6 ± 4.1

**Table 2:** Kinetic fitting parameters to the Korsmeyer-Peppas with lag time drug release model (Equation (2)) of ketoprofen released from PCL-scaffolds obtained by sc-foaming in PBS pH 7.4 medium at 37 °C and 60 rpm.

Scaffold	k (h^n^)	t_lag_ (h)	n	R^2^
PCL(5K)0BA	0.196 ± 0.012	0.454 ± 0.057	0.404 ± 0.019	0.978
PCL(5K)50BA	0.310 ± 0.020	0.499 ± 0.002	0.231 ± 0.017	0.930
PCL(10K)50BA	0.380 ± 0.022	0.499 ± 0.002	0.188 ± 0.015	0.914
PCL(5K)75BA	0.322 ± 0.026	0.500 ± 0.002	0.222 ± 0.021	0.886
PCL(10K)75BA	0.424 ± 0.021	0.500 ± 0.001	0.165 ± 0.013	0.921

## Appendix A

**Table A1:** Thermal properties of PCL-based scaffolds under sc-foaming conditions followed by thermal porogen leaching.

	1st Heating Cycle			2nd Heating Cycle		
Scaffold	T_m_ (°C)	ΔH (J/g)	Crystallinity (%)	T_m_ (°C)	ΔH (J/g)	Crystallinity (%)
PCL(0K)0BA	63.70	84.5	59.5	55.80	60.3	42.5
PCL(5K)50BA	61.74	97.6	72.4	53.02	76.2	56.5
PCL(10K)50BA	61.29	96.1	75.2	52.76	70.4	55.1
PCL(5K)75BA	62.67	100.9	74.8	54.65	75.1	55.7
PCL(10K)75BA	61.74	91.4	71.5	54.72	79.7	62.3
